# *Tetraselmis chuii* Supplementation Increases Skeletal Muscle Nuclear Factor Erythroid 2-Related Factor 2 and Antioxidant Enzyme Gene Expression, and Peak Oxygen Uptake in Healthy Adults: A Randomised Crossover Trial

**DOI:** 10.3390/antiox14040435

**Published:** 2025-04-03

**Authors:** Stuart P. Cocksedge, Carlos Infante, Sonia Torres, Carmen Lama, Lalia Mantecón, Manuel Manchado, Jarred P. Acton, Nehal S. Alsharif, Tom Clifford, Alex J. Wadley, Richard A. Ferguson, Nicolette C. Bishop, Neil R. W. Martin, Stephen J. Bailey

**Affiliations:** 1 School of Sport, Exercise and Health Sciences, Loughborough University, Loughborough LE11 3TU, UK; stuart.cocksedge@coventry.ac.uk (S.P.C.); jarred.acton@loucoll.ac.uk (J.P.A.); nsalsharif@kau.edu.sa (N.S.A.); t.clifford@lboro.ac.uk (T.C.); r.ferguson@lboro.ac.uk (R.A.F.); n.c.bishop@lboro.ac.uk (N.C.B.); n.r.w.martin@lboro.ac.uk (N.R.W.M.); 2 Centre for Physical Activity, Sport and Exercise Sciences, Coventry University, Coventry CV1 5FB, UK; 3 Fitoplancton Marino, S.L., 11500 Cádiz, Spain; carlos.infante@easyalgae.com (C.I.); sonia.torres@easyalgae.com (S.T.); carmen.lama@easyalgae.com (C.L.); emg@easyalgae.com (L.M.); 4 IFAPA Centro El Toruño (Junta de Andalucía), 11500 Cádiz, Spain; manuel.manchado@juntadeandalucia.es; 5 Department of Clinical Nutrition, Faculty of Applied Medical Sciences, King Abdulaziz University, Jeddah 21589, Saudi Arabia; 6 School of Sport, Exercise and Rehabilitation Sciences, College of Life and Environmental Sciences, University of Birmingham, Birmingham B15 2TT, UK; a.j.wadley@bham.ac.uk

**Keywords:** microalgae, redox balance, endurance performance, aerobic fitness

## Abstract

Superoxide dismutase-rich *Tetraselmis chuii* (*T. chuii*) is derived from marine microalgae and has been reported to increase gene expression of nuclear factor erythroid 2-related factor 2 (NRF2) and related antioxidant enzymes in myoblast tissue culture models. Human research has indicated that *T. chuii* supplementation can improve recovery from exercise-induced muscle damage, but its effects on endurance exercise performance and the molecular bases that may underlie any ergogenic effects are unclear. Healthy participants underwent 14 days of supplementation with 25 mg·day^−1^ *T. chuii* and placebo in a randomized, double-blind, crossover experimental design. Prior to and following each supplementation period, participants completed a high-intensity cycling test to assess time to exhaustion and peak oxygen uptake (${\dot{V}O}_{2\mathrm{peak}}$). A resting skeletal muscle biopsy was collected after both supplementation periods to assess gene expression changes. Compared to pre-supplementation values, ${\dot{V}O}_{2\mathrm{peak}}$ was increased following *T. chuii* (*p* = 0.013) but not placebo (*p* = 0.66). Fold-change in glutathione peroxidase 7 [(*GPX7*) 1.26 ± 1.37], glutathione-disulfide reductase [(*GSR*) 1.22 ± 1.41], glutathione S-transferase Mu 3 [(*GSTM3*) 1.34 ± 1.49], peroxiredoxin 6 [(*PRDX6*) 1.36 ± 1.57], extracellular signal-regulated kinase 3 [(*ERK3*) 1.92 ± 2.42], *NRF2* (1.62 ± 2.16), p38 alpha [(*p38a*) 1.33 ± 1.58] and sirtuin 1 [(*SIRT1*) 1.73 ± 2.25] gene expression were higher after *T. chuii* compared to placebo supplementation (*p* < 0.05). Short-term *T. chuii* supplementation increased ${\dot{V}O}_{2\mathrm{peak}}$ and skeletal muscle gene expression of key enzymatic antioxidants (*GPX7*, *GSR*, *GSTM3*, and *PRDX6*), signalling kinases (*ERK3* and *p38a*), post-translational regulators (*SIRT1*), and transcription factors (*NRF2*) that may protect against cellular stress insults.

## 1. Introduction

The ability of the human body to mitigate endogenous and exogenous stressors is required to maintain good health and functional capabilities [1]. When healthy individuals or cells are exposed to such stressors, numerous highly conserved processes can facilitate remodelling towards a phenotype that confers greater resilience to future insults from the same or related stressors through the process of hormesis [2]. One important transcription factor that is activated and orchestrates various adaptive responses upon exposure to cellular stress is nuclear factor erythroid 2-related factor 2 (NRF2) [3]. Indeed, NRF2 controls the expression of more than 200 genes and has been linked to enhancements in a myriad of cellular responses including: detoxication, elimination of damaged macromolecules, and export of xenobiotics; modulating the redox status of the cell by increasing enzymatic antioxidants or synthesizing endogenous reducing agents; regulating apoptosis, cell cycle, and differentiation; elevating chaperones and heat shock proteins; increasing proteins of intercellular adhesion, cytoskeleton, and intracellular transport; regulating ribosomal protein synthesis; bolstering immune and inflammatory responses; and elevating numerous enzymes involved in various aspects of cellular metabolism [4,5]. Accordingly, NRF2 is considered central to cellular homeostasis and the master regulator of the cellular antioxidant response. Data from animal models show that increasing NRF2 activation can enhance exercise capacity [6,7,8], whereas attenuating NRF2 activity compromises exercise capacity [8]. Relatedly, recent data in human skeletal muscle support a role for NRF2 in regulating peak oxygen uptake (${\dot{V}O}_{2\mathrm{peak}}$) and high-intensity exercise performance [9]. Therefore, up-regulating NRF2 activity may confer widespread benefits to local cellular homeostasis, as well as systemic physiological responses and exercise capacity.

In addition to endogenous activation of NRF2, there is great interest in dietary supplements that may activate NRF2, given its potential to improve human health and performance. One supplement reported to transcriptionally activate NRF2 in human myoblasts is superoxide dismutase (SOD)-rich *Tetraselmis chuii* (*T. chuii*). SOD-rich *T. chuii* is a freeze-dried ingredient derived from the green eukaryotic microalgae *T. chuii* (chlorophyte). In addition to SOD, the ingredient contains a number of potentially bioactive compounds such as poly-unsaturated fatty acids (PUFAs), polyphenols, vitamins, carotenoids, and phytosterols [10,11]. When human myoblasts were acutely treated with *T. chuii* for 24 h, *NRF2* gene expression was increased concomitant with elevated gene expression of the enzymatic antioxidants superoxide dismutase (*SOD*), catalase (*CAT*), glutathione peroxidase (*GPx*), and haem oxygenase 1 (*HMOX-1*) [12]. However, whilst these preclinical data were encouraging, only a limited number of gene expression targets were assessed. As such, the potential of *T. chuii* in human skeletal muscle remains unclear, particularly since NRF2 has been implicated in numerous biological processes beyond its role in bolstering the enzymatic antioxidant defense system [4,5]. Using rodent skeletal muscle models, subsequent studies reported that *T. chuii* administration augmented enzymatic antioxidants (SOD, CAT, and GPx) [13] and myogenic factors regulating satellite cell proliferation [14], and attenuated pro-inflammatory cytokines [15] following endurance training compared to endurance training alone and a control condition without endurance training. Clearly, a limitation of these experiments is that the independent effects of *T. chuii* supplementation on skeletal muscle redox and inflammatory responses were not evaluated. Moreover, it is currently unclear whether these findings would be reproduced in human skeletal muscle tissue after dietary *T. chuii* supplementation and, if so, whether this would translate into beneficial effects on systemic physiological responses and exercise capacity. This is a pertinent question, as many purported nutritional NRF2 activators in preclinical models have not been successfully replicated in humans when these nutrients are supplemented [16,17,18].

With regard to the effects of dietary *T. chuii* supplementation on functional responses in humans, most research to date has assessed its effects on recovery of skeletal muscle function following exercise. Specifically, it was reported that short-term supplementation with 25 mg/day *T. chuii* expedited the recovery of skeletal muscle function following muscle-damaging exercise or during periods of non-functional overreaching and intensified resistance training [13,14,15]. By contrast, there is limited research examining the effects of *T. chuii* supplementation on endurance exercise performance and its physiological determinants. In the only study conducted to date, 30 days supplementation with 25 mg/day of *T. chuii* lowered heart rate and increased ${\dot{V}O}_{2\mathrm{peak}}$ [19]. Whilst previous studies have reported beneficial physiological and functional effects after *T. chuii* supplementation [13,14,15,19,20], the molecular bases for any improvements in endurance exercise performance and ${\dot{V}O}_{2\mathrm{peak}}$ [19], as well as post-exercise recovery [13,14,15], have yet to be directly explored in human skeletal muscle tissue. Although reactive oxygen species (ROS) are continually produced by skeletal muscle and other tissues under basal conditions and can serve as beneficial signalling molecules, a series of enzymatic and non-enzymatic antioxidants help to offset the development of dysfunctional redox signalling and oxidative stress [21]. During exercise, ROS production is greatly increased above basal levels, leading to the development of exercise-induced oxidative stress, which has been implicated in fatigue development during exercise [21,22,23]. Therefore, if *T. chuii* supplementation can augment NRF2 signalling and the up-regulation of endogenous antioxidant enzymes, this could enhance exercise capacity by offsetting the development of exercise-induced oxidative stress.

The purpose of the current study was to assess the effect of 14 days supplementation with 25 mg/day SOD-rich *T. chuii* on high-intensity endurance exercise capacity, ${\dot{V}O}_{2\mathrm{peak}}$, and numerous genes related to NRF2 signalling and its downstream effects on antioxidant and inflammatory responses (Table 1) in human skeletal muscle tissue using an OpenArray™ system. It was hypothesised that *T. chuii* supplementation would increase endurance exercise capacity, ${\dot{V}O}_{2\mathrm{peak}}$, and genes related to NRF2 signalling and antioxidant and anti-inflammatory responses in skeletal muscle tissue of healthy adults.

## 2. Materials and Methods

### 2.1. Participant Characteristics

**Figure 1:**
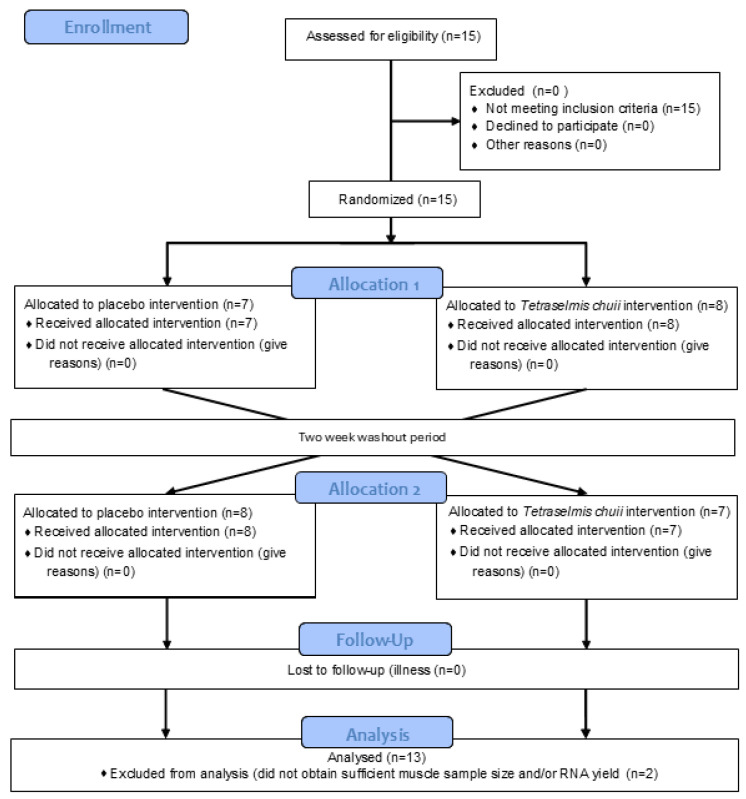
Flow diagram illustrating the study design for trial NCT06822556.

**Figure 2:**
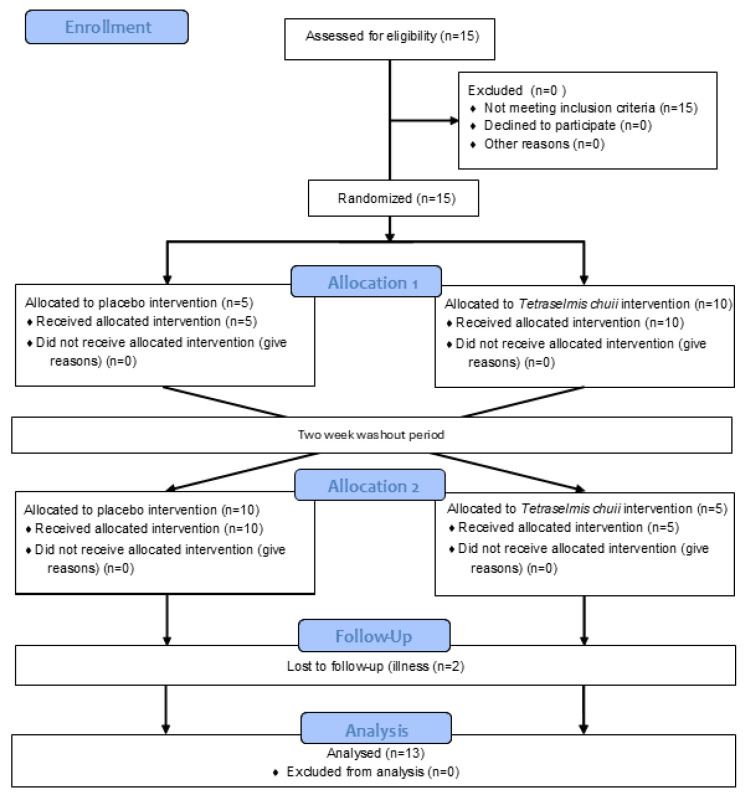
Flow diagram illustrating the study design for trial NCT06831656.

The data presented in the manuscript are from two separate sets of experiments, which recruited two independent groups of human participants. In the study reporting the effects of SOD-rich *T. chuii* supplementation on skeletal muscle gene expression, 15 healthy male participants were recruited, with 13 [mean ± SD (range); age, 22 ± 3 (19–28) years; height, 1.78 ± 0.06 (1.67–1.92) m; body mass, 80 ± 9 (68–96) kg; and BMI, 25 ± 2 (22–29) kg·m^2^] completing the study (Figure 1). This trial was registered with ClinicalTrials.gov ID: NCT06822556. A power calculation was completed using GPower (version 3) using the data of Toro et al. [19]. Based on the increase in ${\dot{V}O}_{2\max}$ reported after *T. chuii* supplementation compared to baseline and assuming a moderate correlation of 0.5 between groups, an effect size of 0.86 was anticipated. With an α of 0.05 and a power of 0.8, a sample size of 13 participants was required, with 15 participants recruited to account for participant withdrawals. In the study reporting the effects of SOD-rich *T. chuii* supplementation on exercise tolerance and physiological responses, 15 healthy male participants were also recruited, with 13 [mean ± SD (range); age, 24 ± 4 (19–34) years; height, 1.81 ± 0.08 (1.69–1.91) m; body mass, 82 ± 9 (69–99) kg; BMI, 25 ± 3 (22–32) kg·m^2^; and ${\dot{V}O}_{2\mathrm{peak}}$, 48 ± 5 (42–59) mL·kg^−1^·min^−1^], completing the study (Figure 2). This trial was registered with ClinicalTrials.gov ID: NCT06831656. A power calculation was completed using GPower (Version 3) using the data of Ramírez et al. [12]. Based on the increase in antioxidant gene expression or enzyme activities reported after *T. chuii* administration compared to baseline and assuming a moderate correlation of 0.5 between groups, an effect size of ≥0.9 was anticipated. With an α of 0.05 and a power of 0.8, a sample size of 12 participants was required, with 15 participants recruited to account for participant withdrawals. All participants were recreationally active, but not highly trained, non-smokers, and were instructed to avoid the consumption of dietary supplements, including antioxidants, and anti-inflammatory medications, including paracetamol and NSAIDs, for the duration of the study. Ethical approval was granted for each set of experiments by Loughborough University Ethics Approvals Committee (Human Participants Sub-Committee) with ethical approval codes of R19-P160 in the study reporting the effects of SOD-rich *T. chuii* supplementation on skeletal muscle gene expression and 2022-6116-8576 in the study reporting the effects of SOD-rich *T. chuii* supplementation on exercise tolerance and physiological responses. Before data collection commenced, each participant received a detailed explanation of the experimental procedures, the associated risks, and the potential benefits, and any questions were addressed. Written informed consent was then obtained from all participants prior to commencing data collection. Prior to all laboratory testing sessions, participants were instructed to arrive at the laboratory in a rested and fully hydrated state, having avoided strenuous exercise and alcohol ingestion for 24 h and caffeine ingestion for 12 h. Participants recorded their dietary intake for the 24 h before the first experimental visit and replicated this diet during the 24 h period preceding subsequent visits. All experimental testing was conducted at the same time of day (±2 h) to minimise the influence of diurnal variations on the measurements.

### 2.2. Experimental Design

In the study reporting the effects of SOD-rich *T. chuii* supplementation on skeletal muscle gene expression, a single resting skeletal muscle biopsy was collected on two occasions over a 6–8-week timeframe. In the study reporting the effects of SOD-rich *T. chuii* supplementation on exercise tolerance and physiological responses, participants reported to the laboratory on 6 occasions over a 10–14-week timeframe. During visit 1, a ramp incremental test was completed for the determination of ${\dot{V}O}_{2\mathrm{peak}}$, gas exchange threshold (GET), and an individualised severe-intensity work rate for use in the subsequent tests. During visit 2, a constant-load step test was completed for familiarisation purposes. During visits 3–6, a constant-load step test was performed for the determination of end-exercise ${\dot{V}O}_{2}$, rating of perceived exertion (RPE), blood lactate concentration (B[La]) and exercise tolerance. Visits 3 and 5 were completed on day 0 whilst visits 4 and 6 were completed on day 14 of each supplementation period. As such, there were 4 experimental conditions: *T. chuii*-Pre, *T. chuii*-Post, placebo (PLA)-Pre (PLA-Pre) and PLA-Post. In both studies, participants were assigned in a double-blind, randomised (using a coin toss), crossover experimental design to receive 2 weeks of dietary supplementation with 25 mg/day SOD-rich *T. chuii* (provided by Fitoplancton Marino, S.L., Spain; a single daily capsule with hemicellulose crystalline as an excipient) or PLA (a single daily capsule containing hemicellulose crystalline only), with supplementation conditions separated by a 2-week washout period. During each supplementation period, participants ingested one capsule on the non-visit days (i.e., days 1–13) in the morning at breakfast, with the visits completed on day 14. The dose and duration of SOD-rich *T. chuii* supplementation used in this study were based on previous studies reporting ergogenic effects with this supplementation strategy [13,14,15,19,20].

### 2.3. Data Collection and Analysis Procedures

#### 2.3.1. Skeletal Muscle Sampling

Skeletal muscle biopsies were obtained from the *m.vastus lateralis* under local anaesthesia (1% lidocaine hydrochloride). After an incision through the skin and underlying fascia site, skeletal muscle tissue was sampled using the Bergstrom-type needle with suction. The samples (~100–250 mg) were minced using scalpels and then submerged in 5× volume RNA*later*™ (ThermoFisher Scientific, Paisley, UK). These samples were stored at 4 °C for 24 h and then transferred to −20 °C prior to later RNA isolation.

#### 2.3.2. RNA Isolation

Muscle lysis was first performed using TRIsure™ (Bioline, London, UK), Lysing Matrix D columns, and a FastPrep™ Lysis System (MP Biomedicals, Santa Ana, CA, USA). Phase separation, using chloroform, was then performed, with the upper aqueous phase recovered for RNA isolation. RNA was then isolated using a spin column procedure. Here, an RNeasy^®^ Mini Kit was used, with on-column DNA digestion using an RNase-free DNase Set and RNA re-purification and concentration using an RNeasy^®^ MinElute^®^ Cleanup Set (Qiagen, Hilden, Germany). The procedure was performed in duplicate per sample and following the manufacturer’s recommended protocol. Sterile conditions were achieved by cleaning the work surfaces and utensils with RNase*Zap*™ RNase Decontamination Solution (Invitrogen, Carlsbad, CA, USA). Furthermore, RNase-free collection tubes and pipette tips were used throughout. The content, concentration, and quality of RNA isolates were determined using a NanoDrop 2000 spectrophotometer (ThermoFisher Scientific, Paisley, UK), with samples attaining an A_260_/A_230_ ratio < 1.8 undergoing a further clean-up kit procedure. RNA isolates were then stored at −80 °C, where they remained until the measurement of gene expression.

#### 2.3.3. OpenArray™ Gene Expression Measurement

Initially, RNA isolates were reverse transcribed using a SuperScript™ VILO™ cDNA Synthesis Kit (ThermoFisher Scientific, Paisley, UK). Here, a master mix was first prepared for each sample, which consisted of 4 μL 5× VILO™ Reaction Mix, 2 μL 10× SuperScript™ Enzyme Mix, 2.5 μg RNA, and diethylpyrocarbonate (DEPC)-treated H_2_O up to a total volume of 20 μL. Following gentle mixing, the cDNA synthesis reaction proceeded by incubation steps, which included 10 min at 25 °C, 60 min at 42 °C, and 5 min at 85 °C. Samples were then stored at −20 °C until the measurement of gene expression.

Gene expression was determined using the QuantStudio™ 12K Flex OpenArray™ System (ThermoFisher Scientific, Paisley, UK), with the 112 × 24 format selected. The genes and their abbreviations and ThermoFisher Scientific Assay ID numbers can be found in Table 1. Targets were selected based on their functions and expression in human skeletal muscle using the Human Protein Atlas database (proteinatlas.org) [24]. *ACTB*, *B2M*, *GAPDH*, *PPP1CA*, and *TBP* were selected as candidate reference genes. 5 μL samples, which consisted of 3.8 μL OpenArray™ PCR MasterMix and 1.2 μL cDNA (20 ng/μL), were first generated. Reactions were then performed in triplicate using 33 nL volumes (~1 ng cDNA) in 384-well Applied Biosystems™ OpenArray™ plates (ThermoFisher Scientific, Paisley, UK). The quantification cycle (C_q_; the cycle number at which the fluorescence level from accumulating amplicons crosses a defined threshold; also known as the threshold cycle, C_t_) was quantified using the relative quantification (C_rq_; also known as the relative threshold, C_rt_) method. Here, the amplification curve is first set to a relative scale that ranges from 0 (minimum fluorescence) to 1 (maximum fluorescence). Using proprietary techniques, a curve that models the reaction efficiency is calculated, and C_q_ is then determined in four steps: (1) an internal reference efficiency level is used to determine the fractional cycle (C_e_) whereby the reaction efficiency curve reaches a predetermined value; (2) the fluorescence level (F_e_) corresponding to the C_e_ is then calculated; (3) the relative fluorescence threshold is computed as a specific percentage of F_e_; and (4) C_q_ is then determined as the cycle number at which the amplification curve crosses the relative fluorescence threshold.

#### 2.3.4. Gene Expression Data Analysis

Gene expression Cq values were obtained using the Thermo Fisher Connect™ online platform and the RQ module (ThermoFisher Scientific, Paisley, UK). Here, the data were first reviewed for quality assurance, with all data represented by a Cq confidence score < 0.8, AMP Score < 1.24, and AMP Status not equal to “AMP” checked and retained only if a valid PCR profile was evident. Also, all data with a replicate SD > 0.5 were checked, and outliers were omitted. The remaining raw data were then exported for further analysis. Here, the data were initially filtered to exclude Cq values < 6 and >28 (the RQ module’s default analysis setting). Also, to build upon the replicate SD check within the RQ module, which can filter errant replicates but not errant samples, the data were filtered based on a range of expected Cq values relative to the condition and participant mean. Here, all data with both a condition and participant SD > 2 were checked (determined by trial and error and intended to omit large errors only), and replicates > ±1 SD from both the condition and participant mean were omitted. Where the review and filtering procedures had removed more than a third of the samples, the gene target was omitted. The replicate Cq values were then averaged, with gene expression (fold-change) determined using the 2^−ΔΔCq^ method [25], with the exception that ΔΔCq was calculated relative to the participant’s control condition ΔCq and not to a group average control condition ΔCq as used in the original method. ΔCq was calculated relative to the geometric mean (GM) of the three most stable reference genes (as determined using the ΔCq method) [26]. GM and geometric standard deviations (GSD) are reported to ensure symmetry for up- and down-regulated genes. Similarly, whilst the data are expressed as 2^−ΔΔCq^, statistical analyses were performed using Log2(2^−ΔCq^) [27]. Finally, the gene expression in the control condition (i.e., PLA) was compared to the difference in gene expression from the control condition to the experimental condition (i.e., ΔPLA-*T. chuii*) to determine if the effect of *T. chuii* was related to basal gene expression.

#### 2.3.5. Exercise Testing Procedures

All exercise tests were performed on an electronically braked (Excalibur Sport) cycle ergometer (Lode, Groningen, The Netherlands), and the saddle and handlebar spatial configuration was recorded during visit 1 and reproduced during the subsequent visits. Participants were first fitted with a facemask to enable the collection of pulmonary gas exchange and ventilation data. All tests began with 5 min of baseline cycling at 50 W. Following this, the work rate increased linearly by 30 W/min during the ramp incremental test (visit 1) or transitioned abruptly to the individualised work rate during the constant-load step test completed in all subsequent visits. Participants cycled at a self-selected cadence (70–90 rpm). All tests continued until the limit of tolerance (Tlim), which was determined as either volitional exhaustion or when the pedal rate dropped by >10 rpm for 5 consecutive s, despite strong verbal encouragement.

For the constant-load step tests, venous blood was collected within 5 min post-exercise from an antecubital forearm vein by the venepuncture technique. Samples were drawn into 6 mL Vacutainer^®^ tubes (Becton-Dickinson Vacutainer, Franklin Lakes, NJ, USA) containing EDTA as the anticoagulant and mixed gently by inversion. Twenty microliters of whole blood was then mixed in 1 mL of Haemolyising Solution (HaB Direct, Southam, UK) in duplicate and stored on ice for same-day B[La] analysis. End-exercise B[La] was measured in duplicate using an automated Biosen C-Line Clinic analyser (EKF Diagnostics, Cardiff, Wales). The analyser was first calibrated using a multi-standard solution tube (HaB Direct, Southam, UK) that contained 12 mmol/L [La].

Breath-by-breath pulmonary gas exchange and ventilation were measured continuously during all exercise tests using the Vyntus™ CPX gas analysis system (CareFusion, Höchberg, Germany). Participants wore a Hans Rudolph 7450 Series V2 Mask and breathed through a flat-fan system that included a digital volume transducer (CareFusion, Höchberg, Germany). The inspired and expired gas volume signal was sampled continuously via a capillary line connected to the flat-fan system. The gas analyser was calibrated immediately before each use with both ambient air and gas of known concentration (16% O_2_ and 5% CO_2_), and the volume transducer was calibrated with a 3 L syringe (Hans Rudolph, Kansas City, MO, USA).

#### 2.3.6. Exercise Physiology and Tolerance Data Analysis Procedures

For the ramp incremental test, the pulmonary gas exchange and ventilation data were exported as consecutive 10 s averages. The ${\dot{V}O}_{2\mathrm{peak}}$ was then denoted as the greatest 30 s rolling average attained before Tlim, whilst GET was determined from a cluster of measurements used previously [28]. These were: (1) the first disproportionate increase in CO_2_ production (${\dot{V}\mathrm{CO}}_{2}$) from visual inspection of individual plots of ${\dot{V}\mathrm{CO}}_{2}$ vs. ${\dot{V}O}_{2}$; (2) an increase in expired ventilation (${\dot{V}}_{E}$)/${\dot{V}O}_{2}$ with no increase in ${\dot{V}}_{E}$/${\dot{V}\mathrm{CO}}_{2}$; and (3) an increase in end-tidal O_2_ tension with no fall in end-tidal CO_2_ tension.

Once the ${\dot{V}O}_{2\mathrm{peak}}$ and GET had been determined, an individualised severe-intensity work rate was calculated. Here, the power outputs associated with the ${\dot{V}O}_{2\mathrm{peak}}$ and GET were first adjusted to account for the ${\dot{V}O}_{2}$ mean response time to ramp incremental exercise (i.e., two-thirds of the ramp rate was subtracted from each power output) [29]. The work rate was then set to 70% of the difference (70%Δ) between these two adjusted power outputs.

For the constant-load step tests, the pulmonary gas exchange and ventilation data were exported as breath-by-breath data. These were first examined to exclude errant breaths caused by coughing, swallowing, sighing, etc., and those values lying more than four standard deviations from the local mean were removed. The data were then linearly interpolated to provide second-by-second values and time-aligned to the start of the step transition. End-exercise ${\dot{V}O}_{2}$ was then denoted as the greatest 30 s rolling average attained before Tlim. All constant-load step tests continued until Tlim, which was determined as either volitional exhaustion or when the pedal rate dropped by >10 rpm for 5 consecutive s. When this occurred, Tlim (s) was noted. For the constant-load step tests, the end-exercise RPE was measured at the Tlim using the Borg 6–20 scale.

### 2.4. Statistical Analysis Procedures

Statistical analyses were performed using SPSS^®^ Statistics version 28 (IBM, New York, NY, USA). All data were first explored for normal distribution using Shapiro-Wilk tests (*p* > 0.05) supported by skewness and kurtosis and visual inspection of histograms. Differences in gene expression between the experimental conditions (PLA and *T. chuii*) were assessed using paired samples *t*-tests (parametric data) or Wilcoxon signed-rank tests (non-parametric data). Differences in end-exercise ${\dot{V}O}_{2}$, RPE and B[La], and Tlim between the experimental conditions (i.e., *T. chuii*-Pre, *T. chuii*-Post, PLA-Pre and PLA-Post) were assessed using two-way repeated-measures ANOVAs [supplement (PLA and *T. chuii*) × time (Pre and Post)] (parametric data) or Friedman’s test (non-parametric data). Additionally, for the two-way repeated-measures ANOVAs only, Greenhouse–Geisser corrections were applied when sphericity was violated, and significant interaction effects were explored further using post hoc paired samples *t*-tests, with family-wise error rate controlled using Holm–Bonferroni corrections. Post hoc effect sizes are presented using Cohen’s dz (parametric data) or Rosenthal’s r (non-parametric data) and interpreted as “small” (0.20–0.49), “moderate” (0.50–0.79), and “large” (≥0.80). Omnibus test effect sizes are presented using Partial Eta Squared (np^2^; parametric data) or Kendall’s w (non-parametric data). Relationships between variables were explored using Pearson’s r (parametric data) or Spearman’s ρ (non-parametric data). Data are presented as the mean ± SD unless stated otherwise. Statistical significance was accepted when *p* < 0.05.

## 3. Results

All participants self-reported 100% compliance in avoiding potentially confounding factors (i.e., not using medication or dietary supplements throughout the study and avoiding strenuous exercise and alcohol ingestion for 24 h, and caffeine ingestion for 12 h before the experimental visits). Furthermore, all participants self-reported consistent dietary habits throughout. The *T. chuii* and PLA supplements were well tolerated, with no side effects reported.

### 3.1. Human Skeletal Muscle Gene Expression

The ΔCq was calculated relative to the geometric mean (GM) of *ACTB*, *B2M* and *PPP1CA* as the three most stable reference genes and subsequently used to calculate Log2(2^−ΔCq^) for statistical analyses of gene expression data.

Expression of 15 genes was up-regulated for PLA-*T. chuii* (ΔPLA-*T. chuii*) with medium to large effect sizes (*p* < 0.05; Table 2). A further 12 genes were up-regulated and 2 genes were down-regulated without statistical significance but with medium effect sizes (*p* = 0.05–0.10, in all instances; Table 2). There were no significant differences in the remaining 66 genes, and effect sizes were small (*p* > 0.05).

Correlation analyses revealed strong negative relationships for PLA-(ΔPLA-*T. chuii*) in 25 genes (*p* < 0.05; Table 3). Moderate negative relationships were revealed in a further 28 genes (*p* < 0.05; Table 3). An additional 23 genes showed moderate negative relationships but without statistical significance (*p* > 0.05). The remaining 18 genes showed weak relationships without statistical significance (*p* > 0.05).

### 3.2. Exercise Physiology and Tolerance

**Figure 3:**
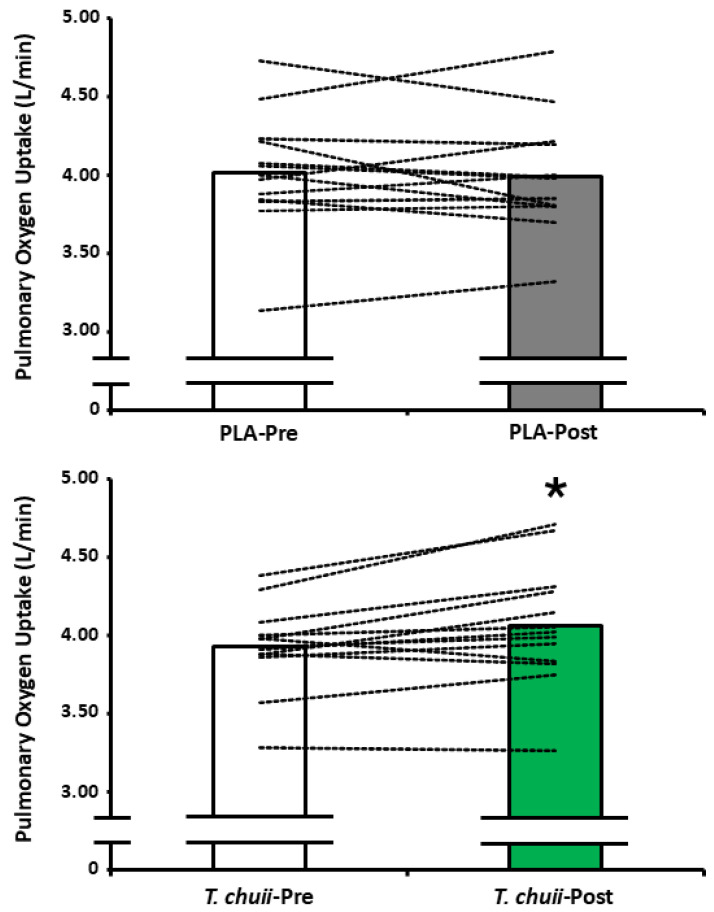
Pulmonary oxygen update during the final 30 s of a high-intensity cycling test continued to exhaustion. Bars represent group mean data whilst dashed lines represent individual participant data. *T. chuii*, 25 mg/day *T. chuii* with hemicellulose crystalline as an excipient; placebo (PLA), PLA containing hemicellulose crystalline only; pre, pre-supplementation baseline; post, post-supplementation for 2 weeks. * denotes significant different (*p* < 0.05) from *T. chuii*-Pre.

There was a supplement × time interaction effect for end-exercise ${\dot{V}O}_{2}$ (*F*_1,12_ = 6.395, *p* = 0.026; *n_p_*^2^ = 0.348), but no main effect for time (*F*_1,12_ = 1.927, *p* = 0.190; *n_p_*^2^ = 0.138) or supplement (*F*_1,12_ = 0.270, *p* = 0.613; *n_p_*^2^ = 0.022). Post hoc paired samples *t*-tests revealed that end-exercise ${\dot{V}O}_{2}$ was greater for *T. chuii*-Post compared to *T. chuii*-Pre (*p* = 0.013; *dz* = 0.8; Table 4; Figure 3). End-exercise ${\dot{V}O}_{2}$ was not different between PLA-Pre and PLA-Post (*p* = 0.66; *dz* = 0.1) and was not different between PLA-Pre and *T. chuii*-Pre (*p* = 0.06; *dz* = 0.6) or PLA-Post and *T. chuii*-Post (*p* = 0.11; *dz* = 0.5). Friedman’s test revealed that end-exercise RPE was unchanged (*p* = 0.34; *w* = 0.1; Table 4). There was a main effect for time (*F*_1,12_ = 9.238, *p* = 0.013; *n_p_*^2^ = 0.412), but no main effect for supplement (*F*_1,12_ = 0.877, *p* = 0.367; *n_p_* =0.068) and no supplement × time interaction effect (*F*_1,12_ = 0.026, *p* = 0.847; *n_p_*^2^ = 0.002; Table 4) for end-exercise B[La]. Similarly, there was a main effect for time (*F*_1,12_ = 9.238, *p* = 0.010; *n_p_*^2^ = 0.435), but no main effect for supplement (*F*_1,12_ = 0.270, *p* = 0.613; *n_p_*^2^ = 0.022) and no supplement × time interaction effect (*F*_1,12_ = 0.692, *p* = 0.422; *n_p_*^2^ = 0.055) (Table 4) for Tlim.

## 4. Discussion

The principal original findings of the current study were that 2 weeks of *T. chuii* supplementation increased gene expression of *NRF2*, antioxidant enzymes (*GPX7*, *GSR*, *GSTM3* and *PRDX6*), signalling kinases (*ERK3* and *p38a*) and post-translational regulators (*SIRT1*) in human skeletal muscle tissue. Moreover, the magnitude to which *T. chuii* supplementation increased gene expression related to NRF2 signalling, transcription factors, redox balance and inflammation was inversely related to baseline gene expression; that is, those individuals that typically presented with the smallest basal gene expression exhibited a greater increase in gene expression following *T. chuii* supplementation. Supplementation with *T. chuii* also increased end-exercise ${\dot{V}O}_{2}$ during an exhaustive severe-intensity exercise test. These observations are broadly consistent with the experimental hypotheses and suggest that *T. chuii* supplementation can positively modulate *NRF2* and antioxidant enzyme gene expression in skeletal muscle and increase ${\dot{V}O}_{2\mathrm{peak}}$ in healthy adults. As such, *T. chuii* supplementation has the potential to confer beneficial effects on local skeletal muscle signalling and functional systemic physiological processes.

Whilst increased *NRF2* gene expression has previously been reported in human myoblasts after *T. chuii* treatment [12], an important novel contribution of the current study is the observation that short-term dietary *T. chuii* supplementation increased *NRF2* gene expression in human muscle tissue. A further important expansion of the data presented in the current manuscript compared to the previous paper by Ramírez and colleagues [12] is the use of OpenArray™ to assess expression of a far greater number of genes relating to redox and inflammatory signalling as well as transcripts with the potential to modulate NRF2 signalling. Principally, NRF2 is recognised as the master regulator of the cellular antioxidant response [30]. Accordingly, *T. chuii* supplementation increased skeletal muscle gene expression of the enzymatic antioxidants *GPX7* and *PRDX6* [31,32], which are integral to the elimination of the ROS, hydrogen peroxide (H_2_O_2_) [33]. GPX7 is an important sensor of oxidative stress and confers antioxidant effects indirectly through increasing protein activity of downstream targets [31]. On the other hand, PRDX6 can directly reduce H_2_O_2_ to water using reduced glutathione (GSH) as the reducing substrate, forming oxidised glutathione (GSSG) [32]. In addition, *T. chuii* supplementation increased *GSR* gene expression, which is important to sustain the flux of H_2_O_2_ reduction through PRDX6, as well as some other enzymatic antioxidants that use GSH as a reduction substrate (e.g., GPX and TXN), as GSR catalyses the reduction of GSSG of GSH [34]. There was also an increase in *GSTM3* gene expression after *T. chuii* supplementation in the current study. Hydroperoxides are ROS that can be detoxified by PRDX6 [32] and via biotransformation (i.e., conjugation with GSH) by GSTM3 in the cytosol [35,36]. Therefore, the increases in skeletal muscle *GPX7*, *PDRX6*, *GSR* and *GSTM3* gene expression after *T. chuii* supplementation indicates that muscle may be better equipped with enzymatic antioxidants to preserve a more optimal redox balance when challenged by pro-oxidative insults.

An interesting observation of the current study is an increase in skeletal muscle gene expression of *PLA2G12A* and *PLA2G16* after *T. chuii* supplementation. Since PLA2 is recognised as a direct source of ROS in skeletal muscle and can indirectly increase ROS production through activation of nicotinamide adenine dinucleotide phosphate oxidase (NADPH) oxidases (NOX) [37], an increase in pro-oxidant enzymes (*PLA2G12A* and *PLA2G16*) alongside increases in antioxidant enzymes (*GPX7*, *PDRX6*, *GSR* and *GSTM3*) gene expression after *T. chuii* supplementation appears somewhat paradoxical. However, a physiological and low-dose increase in ROS production can increase NRF2 signalling to promote an up-regulation in endogenous antioxidant enzymes, consistent with the principle of hormetic nutrition, as reported with other purported dietary antioxidants such as (poly)phenols [38,39]. Increased skeletal muscle *PLA2G12A* and *PLA2G16* may also have contributed to the activation of the redox-sensitive transcripts, *ERK3* (*MAPK6*) and *p38a* (*MAPK14*), which contribute to the regulation of enzymatic antioxidants [40,41]. There were also increases in *JUN* and *SIRT1* gene expression after *T. chuii* supplementation, which can aid NRF2 signalling. Indeed, JUN can dimerise with NRF2 to augment antioxidant response element (ARE)-mediated increases in expression of enzymatic antioxidants [42,43,44]. In addition, SIRT1 is positively associated with NRF2 content and deacetylates NRF2, which increases the stability and transcriptional activity of NRF2 and stimulates the transport of NRF2 to the nucleus to promote ARE effects [45]. Whilst these gene expression changes may collectively indicate that skeletal muscle NRF2 signalling was increased, *CUL3* expression was also increased following *T. chuii* supplementation, with CUL3 being part of the KEAP1-CUL3-RBX1 E3 ubiquitin ligase complex which targets NRF2 for ubiquitination and proteasomal degradation [46,47]. Increased *CUL3* expression, therefore, indicates a potential negative feedback response following an increase in *NRF2* gene expression after *T. chuii* supplementation to modulate production of enzymatic antioxidants, which could promote an overly reduced cellular redox balance and compromise aspects of skeletal muscle function [48]. However, there is also evidence that CUL3 can ubiquitinate and subsequently degrade KEAP1 [49], which could aid NRF2 migration to the nucleus and up-regulate enzymatic antioxidants. Therefore, in aggregate, the gene expression data imply that muscle NRF2 signalling and antioxidant enzymes may have been increased after short-term *T. chuii* supplementation. These observations are important since previous purported nutritional NRF2 activators have largely shown no effect in human nutritional trials [16,17,18].

As well as increasing skeletal muscle gene expression of enzymatic antioxidants and associated signalling cascades, short-term *T. chuii* supplementation increased ${\dot{V}O}_{2\mathrm{peak}}$. This observation is consistent with a previous study which reported increased ${\dot{V}O}_{2\mathrm{peak}}$ after 30 days of supplementation with 25 mg/day *T. chuii* [19]. Our findings expand this previous observation by indicating that a shorter duration of *T. chuii* supplementation can also increase ${\dot{V}O}_{2\mathrm{peak}}$. The concomitant increases in ${\dot{V}O}_{2\mathrm{peak}}$ and gene expression of *NRF2* and enzymatic antioxidants are also consistent with previous reports suggesting that these variables are positively associated [9,50]. In addition to its role as a regulator of antioxidant responses, NRF2 has been implicated in mitochondrial biogenesis [51]. Moreover, SIRT1 and MAPKs are also recognised transcripts to orchestrate mitochondrial biogenesis [52]. It is possible, therefore, that the increased ${\dot{V}O}_{2\mathrm{peak}}$ after *T. chuii* supplementation could have been mediated by increased mitochondrial biogenesis. Whilst PGC1α is considered to be the master regulator of mitochondrial biogenesis and was not increased after *T. chuii* supplementation in the current study, alternative signalling mechanisms can contribute to mitochondrial biogenesis, including NRF2, SIRT1 and MAPKs, which were elevated after *T. chuii* supplementation in this study [52,53]. Whether NRF2, SIRT1 and MAPKs can increase mitochondrial biogenesis independent of PGC1α is unclear. It is also possible that gene expression of alternative transcription factors could have been increased after *T. chuii* supplementation in the current study and could have potentially elicited mitochondrial biogenesis via PGC1α-dependent or PGC1α-independent mechanisms. Further research is required to explore these possibilities. It is recognised that the lack of assessment of mitochondrial respiration and content markers is a limitation of this study. We also cannot exclude the possibility that increased ${\dot{V}O}_{2\mathrm{peak}}$ after *T. chuii* supplementation could have been linked to improved skeletal muscle perfusion or that the supplement elicited effects in different organs to skeletal muscle, but further research is required to assess these possibilities. It is also acknowledged that since skeletal muscle gene expression and ${\dot{V}O}_{2\mathrm{peak}}$ were assessed in two separate studies after *T. chuii* supplementation, this compromises direct comparison of the link between these responses in the current manuscript. Further research is also required to assess whether *T. chuii* supplementation can improve other cardiorespiratory variables during exercise, such as ventilatory efficiency (${\dot{V}}_{E}$/${\dot{V}\mathrm{CO}}_{2}$) [54]. The increase in ${\dot{V}O}_{2\mathrm{peak}}$ is of functional significance since it is positively associated with exercise capacity and performance [55] and inversely associated with ill health and mortality [56,57]. Given the increase in ${\dot{V}O}_{2\mathrm{peak}}$, it is surprising, therefore, that *T. chuii* supplementation did not significantly increase Tlim in the current study. It is possible that we were statistically underpowered to detect this 17% increase in Tlim compared to baseline and, as such, further research is required to assess the ergogenic potential of *T. chuii* supplementation in different exercise settings. It is also possible that, since PLA elicited a non-significant increase in Tlim of 11% compared to baseline, there may have been an insufficient washout between conditions for participants who underwent the *T. chuii* supplementation arm of the study first.

Whilst the current study observed increases in skeletal muscle gene expression of key enzymatic antioxidants (*GPX7*, *GSR*, *GSTM3*, and *PRDX6*), signalling kinases (*ERK3* and *p38a*), post-translational regulators (*SIRT1*), and transcription factors (*NRF2*), a limitation of the current study is that proteomic analyses were not completed to determine whether these transcriptional responses translated into increased protein expression. Further research is therefore required to probe the molecular bases by which *T. chuii* supplementation can improve cell signalling in skeletal muscle, and other tissues, alongside the potential for wider health benefits. It is also recognised that, since the participants in the current study were all healthy and active, the findings of the current study may not translate to different populations. It is of interest to determine whether individuals presenting with a more pro-oxidative or proinflammatory state, such as older adults or clinical populations, would benefit more from NRF2 activation and associated antioxidant responses that may ensue following *T. chuii* supplementation. Indeed, participants presenting with depleted antioxidant status appear more likely to improve exercise capacity and skeletal muscle function after dietary antioxidant supplementation [58]. Consistent with this, there was an inverse correlation between basal expression of 53 genes and the magnitude of increase in these genes after *T. chuii* supplementation. Moreover, some of the skeletal muscle gene expression changes reported herein may improve understanding of the molecular bases for improved post-exercise recovery reported in previous studies after the same *T. chuii* supplementation protocol administered herein [13,14,15].

## 5. Conclusions

Short-term supplementation with *T. chuii* increased skeletal muscle gene expression of *NRF2*, positive NRF2 signalling mediators (*JUN* and *SIRT1*) and antioxidant enzymes (*GPX7*, *PDRX6*, *GSR* and *GSTM3*). *T. chuii* therefore appears to hold promise as a nutritional NRF2 signalling and antioxidant precursor, at least in healthy human skeletal muscle. In addition, *T. chuii* supplementation increased ${\dot{V}O}_{2\mathrm{peak}}$ and since the supplement also increased skeletal muscle *NRF2*, *MAPK*, and *SIRT1* gene expression, which are key mediators of mitochondrial biogenesis, the increased ${\dot{V}O}_{2\mathrm{peak}}$ could be a function of enhanced mitochondrial capacity. Therefore, these data suggest *T. chuii* may represent a natural and blue-food supplement with the potential to improve skeletal muscle antioxidant responses and systemic oxidative metabolism in healthy adult humans.

## Figures and Tables

**Table 1:** Gene names, gene symbols and ThermoFisher Scientific Assay IDs for human skeletal muscle OpenArray™ gene expression analyses.

Gene Name	Gene Symbol	ThermoFisher Scientific Assay ID
**Cellular Response to Chemical Stress**		
Catalase	*CAT*	Hs00156308_m1
Ferritin Light Chain	*FTL*	Hs00830226_gH
Glutamate-Cysteine Ligase Catalytic Subunit	*GCLC*	Hs00155249_m1
Glutamate-Cysteine Ligase Modifier Subunit	*GCLM*	Hs00978072_m1
Glutaredoxin	*GLRX*	Hs00829752_g1
Glutathione Peroxidase 1	*GPX1*	Hs00829989_gH
Glutathione Peroxidase 3	*GPX3*	Hs01078668_m1
Glutathione Peroxidase 4	*GPX4*	Hs00989766_g1
Glutathione Peroxidase 7	*GPX7*	Hs04194449_s1
Glutathione Disulfide Reductase	*GSR*	Hs00167317_m1
Heme Oxygenase 1	*HMOX1*	Hs01110250_m1
Heme Oxygenase 2	*HMOX2*	Hs01558390_m1
Kelch-Like ECH Associated Protein 1	*KEAP1*	Hs00202227_m1
Nuclear Factor, Erythroid 2 Like 2	*NRF2*	Hs00975961_g1
Peroxiredoxin 1	*PRDX1*	Hs00602020_mH
Peroxiredoxin 2	*PRDX2*	Hs00853603_s1
Peroxiredoxin 3	*PRDX3*	Hs00428953_g1
Peroxiredoxin 4	*PRDX4*	Hs01056076_m1
Peroxiredoxin 5	*PRDX5*	Hs00738905_g1
Peroxiredoxin 6	*PRDX6*	Hs00705355_s1
Superoxide Dismutase 1	*SOD1*	Hs00533490_m1
Superoxide Dismutase 2	*SOD2*	Hs00167309_m1
Superoxide Dismutase 3	*SOD3*	Hs04973910_s1
Sulfiredoxin 1	*SRXN1*	Hs00607800_m1
Thioredoxin 2	*TXN2*	Hs00429399_g1
Thioredoxin Reductase 1	*TXNRD1*	Hs00917067_m1
Thioredoxin Reductase 2	*TXNRD2*	Hs00272352_m1
**Cellular Source of Chemical Stress**		
Phospholipase A2 Group XIIA	*PLA2G12A*	Hs04976755_s1
Phospholipase A2 Group XV	*PLA2G15*	Hs00202399_m1
Phospholipase A2 Group XVI	*PLA2G16*	Hs00912734_m1
Phospholipase A2 Group IIA	*PLA2G2A*	Hs00179898_m1
Phospholipase A2 Group IVC	*PLA2G4C*	Hs01003754_m1
Phospholipase A2 Group IVF	*PLA2G4F*	Hs02577398_m1
**Biological Oxidations**		
Glutathione S-Transferase Kappa 1	*GSTK1*	Hs01114170_m1
Glutathione S-Transferase Mu 1	*GSTM1*	Hs01683722_gH
Glutathione S-Transferase Mu 2	*GSTM2*	Hs00265266_g1
Glutathione S-Transferase Mu 3	*GSTM3*	Hs00356079_m1
Glutathione S-Transferase Mu 4	*GSTM4*	Hs00426432_m1
Glutathione S-Transferase Mu 5	*GSTM5*	Hs00757076_m1
Glutathione S-Transferase Omega 1	*GSTO1*	Hs02383465_s1
Glutathione S-Transferase Pi 1	*GSTP1*	Hs00943350_g1
Glutathione S-Transferase Theta 2	*GSTT2*	Hs04942283_g1
Microsomal Glutathione S-Transferase 3	*MGST3*	Hs01058946_m1
NAD(P)H Quinone Dehydrogenase 1	*NQO1*	Hs01045993_g1
NAD(P)H Quinone Dehydrogenase 2	*NQO2*	Hs01056950_m1
**Signalling Pathways**		
C-C Motif Chemokine Ligand 2	*CCL2*	Hs00234140_m1
Extracellular Signal-Regulated Kinase 1	*ERK1 (MAPK3)*	Hs00385075_m1
Extracellular Signal-Regulated Kinase 2	*ERK2 (MAPK1)*	Hs01046830_m1
Extracellular Signal-Regulated Kinase 3	*ERK3 (MAPK6)*	Hs00833126_g1
Extracellular Signal-Regulated Kinase 5	*ERK5 (MAPK7)*	Hs00611114_g1
Interleukin 10	*IL10*	Hs00961622_m1
Interleukin 18	*IL18*	Hs01038788_m1
Interleukin 1 Receptor Antagonist	*IL1RN*	Hs00893626_m1
Interleukin 6	*IL6*	Hs00174131_m1
Nuclear Factor Kappa B Subunit 1	*NFKB1*	Hs00765730_m1
Nuclear Factor Kappa B Subunit 2	*NFKB2*	Hs01028890_g1
NLR Family Pyrin Domain Containing 3	*NLRP3*	Hs00918082_m1
p38 Alpha	*p38a (MAPK14)*	Hs01051152_m1
p38 Gamma	*p38g (MAPK12)*	Hs00268060_m1
Sirtuin 1	*SIRT1*	Hs01009006_m1
Tumor Necrosis Factor Alpha	*TNFa*	Hs00174128_m1
**Antioxidant Response Elements**		
Activating Transcription Factor 1	*ATF1*	Hs00909673_m1
Activating Transcription Factor 4	*ATF4*	Hs00909569_g1
BTB Domain and CNC Homolog 1	*BACH1*	Hs00230917_m1
BTB Domain and CNC Homolog 2	*BACH2*	Hs00935338_m1
BTB Domain Containing 1	*BTBD1*	Hs00228832_m1
BTB Domain Containing 10	*BTBD10*	Hs00900692_g1
BTB Domain Containing 2	*BTBD2*	Hs00215064_m1
BTB Domain Containing 6	*BTBD6*	Hs00746410_s1
BTB Domain Containing 7	*BTBD7*	Hs01568248_m1
BTB Domain Containing 9	*BTBD9*	Hs04987111_m1
FOS Proto-Oncogene	*FOS*	Hs04194186_s1
FOS Like 1	*FOSL1*	Hs00759776_s1
FOS Like 2	*FOSL2*	Hs01050117_m1
Forkhead Box O3	*FOXO3*	Hs00818121_m1
JUN Proto-Oncogene	*JUN*	Hs01103582_s1
JUNB Proto-Oncogene	*JUNB*	Hs00357891_s1
JUND Proto-Oncogene	*JUND*	Hs04187679_s1
Kruppel Like Factor 2	*KLF2*	Hs07291763_gH
MAF bZip Transcription Factor	*MAF*	Hs04185012_s1
MAF bZip Transcription Factor A	*MAFA*	Hs04419852_s1
MAF bZip Transcription Factor B	*MAFB*	Hs00534343_s1
MAF bZip Transcription Factor F	*MAFF*	Hs05026540_g1
MAF bZip Transcription Factor G	*MAFG*	Hs00536278_s1
PPARG Coactivator 1 Alpha	*PGC1a*	Hs00173304_m1
**Cell Fate**		
Apoptosis Inducing Factor	*AIFM1*	Hs00377585_m1
BCL2 Antagonist/Killer 1	*BAK1*	Hs00832876_g1
BCL2 Associated X	*BAX*	Hs00180269_m1
BCL2 Apoptosis Regulator	*BCL2*	Hs04986394_s1
BH3 Interacting Domain Death Agonist	*BID*	Hs00609632_m1
Caspase 1	*CASP1*	Hs00354836_m1
Caspase 10	*CASP10*	Hs01017899_m1
Caspase 3	*CASP3*	Hs00234387_m1
Caspase 8	*CASP8*	Hs06630780_s1
CASP8 and FADD Like Apoptosis Regulator	*CFLAR*	Hs01116280_m1
Tumor Protein p53	*TP53*	Hs01034249_m1
**Miscellaneous**		
Apurinic/Apyrimidinic Endodeoxyribonuclease 1	*APEX1*	Hs00172396_m1
Calpain 1	*CAPN1*	Hs00559804_m1
Calpain 3	*CAPN3*	Hs00544982_m1
Cullin 3	*CUL3*	Hs00180183_m1
Metallothionein 2A	*MT2A*	Hs02379661_g1
N-Terminal EF-Hand Calcium Binding Protein 1	*Necab1*	Hs00242747_m1
Nitric Oxide Synthase 2	*NOS2*	Hs01075529_m1
Nitric Oxide Synthase 3	*NOS3*	Hs01574665_m1
Sequestosome 1	*SQSTM1*	Hs01061917_g1
Uncoupling Protein 2	*UCP2*	Hs01075227_m1
Vascular Endothelial Growth Factor A	*VEGFA*	Hs00900055_m1
**Reference Gene Candidates**		
Actin Beta	*ACTB*	Hs01060665_g1
Beta 2 Microglobulin	*B2M*	Hs00187842_m1
Glyceraldehyde-3-Phosphate Dehydrogenase	*GAPDH*	Hs02786624_g1
Protein Phosphatase 1 Catalytic Subunit Alpha	*PPP1CA*	Hs00267568_m1
TATA-Box Binding Protein	*TBP*	Hs00427620_m1

**Table 2:** Gene expression in RNA isolated from resting human skeletal muscle following dietary supplementation for 2 weeks with 25 mg/day *T. chuii* with hemicellulose crystalline as an excipient compared to a placebo containing hemicellulose crystalline only.

Gene Name	Gene Symbol	2^−ΔΔCq^ (GM ± GSD)	*t*	*p* *w*	*dz*	ES *r*
**PLA-*****T. chuii*** **(*p* < 0.05, Medium to Large ES)**						
BTB domain containing 7	*BTBD7*	1.38 ± 1.62	0.03		0.7	
Caspase 10	*CASP10*	1.34 ± 1.59	0.04		0.6	
C-C Motif Chemokine Ligand 2	*CCL2*	1.39 ± 1.39	0.00		1.0	
Cullin 3	*CUL3*	1.77 ± 2.49	0.04		0.6	
Extracellular Signal-Regulated Kinase 3	*ERK3 (MAPK6)*	1.92 ± 2.42	0.02		0.7	
Glutathione Peroxidase 7	*GPX7*	1.26 ± 1.37	0.02		0.7	
Glutathione-Disulfide Reductase	*GSR*	1.22 ± 1.41		0.05		0.6
Glutathione S-Transferase Mu 3	*GSTM3*	1.34 ± 1.49	0.02		0.7	
JUN Proto-Oncogene	*JUN*	1.53 ± 1.89	0.03		0.7	
Nuclear Factor, Erythroid 2 Like 2	*NRF2*	1.62 ± 2.16		0.03		0.6
p38 Alpha	*p38a (MAPK14)*	1.33 ± 1.58	0.04		0.6	
Phospholipase A2 Group XIIA	*PLA2G12A*	1.63 ± 2.05	0.03		0.7	
Phospholipase A2 Group XVI	*PLA2G16*	1.36 ± 1.42	0.01		0.9	
Peroxiredoxin 6	*PRDX6*	1.36 ± 1.57	0.03		0.7	
Sirtuin 1	*SIRT1*	1.73 ± 2.25	0.03		0.7	
**PLA-*****T. chuii*** **(*p* > 0.05, *p* < 0.1, Medium ES)**						
Apoptosis Inducing Factor	*AIFM1*	1.49 ± 2.14	0.08		0.5	
Activating Transcription Factor 1	*ATF1*	1.54 ± 2.30	0.09		0.5	
BTB Domain and CNC Homolog 1	*BACH1*	1.61 ± 2.39	0.07		0.5	
BTB domain containing 1	*BTBD1*	1.37 ± 1.79	0.08		0.5	
Calpain 3	*CAPN3*	1.29 ± 1.53	0.05		0.6	
Caspase 8	*CASP8*	1.44 ± 1.95	0.07		0.5	
Catalase	*CAT*	1.40 ± 1.87	0.07		0.5	
Extracellular Signal-Regulated Kinase 2	*ERK2 (MAPK1)*	1.32 ± 1.60	0.06		0.6	
Extracellular Signal-Regulated Kinase 5	*ERK5 (MAPK7)*	0.87 ± 1.27	0.05		0.6	
Glutamate-Cysteine Ligase Modifier Subunit	*GCLM*	1.38 ± 1.78	0.07		0.6	
Interleukin 18	*IL18*	2.05 ± 3.29	0.05		0.6	
Peroxiredoxin 3	*PRDX3*	1.30 ± 1.56	0.06		0.6	
Superoxide Dismutase 2	*SOD2*	1.40 ± 1.86	0.08		0.5	

2^−ΔΔCq^, fold-change (gene expression); GM, geometric mean; GSD, geometric standard deviation; *p*, significance value; *t*, paired samples *t*-test significance value; *w*, Wilcoxon signed-rank test significance value; ES, effect size; *dz*, Cohen’s effect size; *r*, Rosenthal’s effect size; placebo (PLA), dietary supplementation for 2 weeks with a PLA containing hemicellulose crystalline only; *T. chuii*, dietary supplementation for 2 weeks with 25 mg/day of the ingredient with hemicellulose crystalline as an excipient; PLA-*T. chuii*, *T. chuii* compared to PLA. Green highlight indicates up-regulation, and red highlight indicates down-regulation. The shading represents a 3-colour scale (i.e., green, white, and red), which centres on 1-fold expression (i.e., no change) with the minimum and maximum set to 0.5-fold (i.e., half) and 2-fold (i.e., double) expression, respectively.

**Table 3:** Correlations between gene expression in RNA isolated from resting human skeletal muscle following dietary supplementation for 2 weeks with a placebo containing hemicellulose crystalline only compared to the difference in gene expression between placebo and dietary supplementation for 2 weeks with 25 mg/day *T. chuii* with hemicellulose crystalline as an excipient.

Gene Name	Gene Symbol	*r*	*CC* *p*
**PLA-(ΔPLA-** ***T. chuii*** **) (*p* < 0.05, Strong CC)**			
Apoptosis Inducing Factor	*AIFM1*	−0.8	<0.01
Apurinic/Apyrimidinic Endodeoxyribonuclease 1	*APEX1*	−0.8	<0.01
Activating Transcription Factor 1	*ATF1*	−0.9	<0.01
BTB Domain and CNC Homolog 1	*BACH1*	−0.8	<0.01
Bcl2 Apoptosis Regulator	*BCL2*	−0.9	<0.01
BTB Domain Containing 2	*BTBD2*	−0.8	<0.01
BTB Domain Containing 9	*BTBD9*	−0.8	<0.01
Caspase 1	*CASP1*	−0.9	<0.01
Caspase 3	*CASP3*	−0.9	<0.01
Caspase 8	*CASP8*	−0.8	<0.01
Catalase	*CAT*	−0.8	<0.01
Cullin 3	*CUL3*	−0.9	<0.01
Extracellular Signal-Regulated Kinase 3	*ERK3 (MAPK6)*	−0.8	<0.01
Forkhead Box O3	*FOXO3*	−0.8	<0.01
Lipoxygenase Homology Domains 1	*LOXHD1*	−0.8	<0.01
MAF bZip Transcription Factor G	*MAFG*	−0.8	<0.01
Microtubule Associated Protein Tau	*MAPT*	−0.9	<0.01
N-Terminal EF-Hand Calcium Binding Protein 1	*Necab1*	−0.8	<0.01
Nuclear Factor Kappa B Subunit 1	*NFKB1*	−0.9	<0.01
PPARG Coactivator 1 Alpha	*PGC1a*	−0.9	<0.01
Phospholipase A2 Group XIIA	*PLA2G12A*	−0.8	<0.01
Peroxiredoxin 3	*PRDX3*	−0.8	<0.01
Superoxide Dismutase 2	*SOD2*	−0.8	<0.01
Thioredoxin Reductase 1	*TXNRD1*	−0.9	<0.01
Thioredoxin Reductase 2	*TXNRD2*	−0.8	<0.01
**PLA-(ΔPLA-** ***T. chuii*** **) (*p* < 0.05, Moderate CC)**			
BCL2 Antagonist/Killer 1	*BAX*	−0.7	<0.01
BCL2 Antagonist/Killer 1	*BAK1*	−0.6	0.03
Calpain 3	*CAPN3*	−0.6	0.02
Extracellular Signal-Regulated Kinase 5	*ERK5 (MAPK7)*	−0.7	0.01
FOS Like 2	*FOSL2*	−0.7	0.01
Ferritin Light Chain	*FTL*	−0.6	0.04
Glutamate-Cysteine Ligase Modifier Subunit	*GCLM*	−0.7	0.01
Glutaredoxin	*GLRX*	−0.6	0.04
Glutathione Peroxidase 1	*GPX1*	−0.7	0.01
Glutathione S-Transferase Mu 4	*GSTM4*	−0.6	0.05
Glutathione S-Transferase Pi 1	*GSTP1*	−0.6	0.02
Heme Oxygenase 2	*HMOX2*	−0.6	0.02
Interleukin 18	*IL18*	−0.7	0.01
JUND Proto-Oncogene	*JUND*	−0.7	0.01
Kruppel Like Factor 2	*KLF2*	−0.7	0.01
MAF bZip Transcription Factor	*MAF*	−0.7	0.01
MAF bZip Transcription Factor B	*MAFB*	−0.6	0.04
Microsomal Glutathione S-Transferase 3	*MGST3*	−0.7	0.00
Nitric Oxide Synthase 3	*NOS3*	−0.7	0.01
Nuclear Factor Kappa B Subunit 2	*NFKB2*	−0.7	0.01
P38 Gamma	*p38g (MAPK12)*	−0.7	0.01
Phospholipase A2 Group XV	*PLA2G15*	−0.7	0.02
Phospholipase A2 Group IVC	*PLA2G4C*	−0.7	0.01
Peroxiredoxin 5	*PRDX5*	−0.6	0.03
Peroxiredoxin 6	*PRDX6*	−0.6	0.03
Sequestosome 1	*SQSTM1*	−0.6	0.03
Sirtuin 1	*SIRT1*	−0.7	0.00
Superoxide Dismutase 3	*SOD3*	−0.7	0.01
Thioredoxin 2	*TXN2*	−0.7	0.00

CC, correlation coefficient; r, Pearson’s correlation coefficient; *p*, significance value; placebo (PLA), dietary supplementation for 2 weeks with a PLA containing hemicellulose crystalline only; *T. chuii*, dietary supplementation for 2 weeks with 25 mg/day *T. chuii* with hemicellulose crystalline as an excipient; Δ, difference; PLA-(ΔPLA-*T. chuii*), PLA compared to the difference between PLA and *T. chuii*.

**Table 4:** End-exercise pulmonary oxygen uptake, rating of perceived exertion, blood lactate concentration, and exercise tolerance following a step increment in work rate from baseline to severe-intensity cycling prior to and following dietary supplementation for 2 weeks with *T. chuii* with hemicellulose crystalline as an excipient and a placebo containing hemicellulose crystalline only.

	PLA-Pre	PLA-Post	*T. chuii*-Pre	*T. chuii*-Post
End-exercise ${\dot{V}O}_{2}$ (L/min)	4.02 ± 0.38	3.99 ± 0.37	3.92 ± 0.28	4.06 ± 0.38 *
End-exercise RPE (AU)	20 ± 0	20 ± 1	20 ± 1	20 ± 0
End-exercise B[La] (mmol/L)	10.46 ± 2.17	11.02 ± 1.34	10.85 ± 1.92	11.52 ± 1.91
Tlim (s)	410 ± 80	456 ± 134	411 ± 82	480 ± 124

Data are presented as mean ± SD. *T. chuii*, 25 mg/day *T. chuii* with hemicellulose crystalline as an excipient; placebo (PLA), PLA containing hemicellulose crystalline only; pre, pre-supplementation baseline; post, post-supplementation for 2 weeks; RPE, rating of perceived exertion; AU, arbitrary units; B[La], blood lactate concentration; mmol/L, millimoles per litre; Tlim, limit of tolerance; s, seconds; ${\dot{V}O}_{2}$, pulmonary oxygen uptake. * denotes significantly different from *T. chuii*-Pre, *p* < 0.05.

## Data Availability

The original contributions presented in this study are included in the article/Supplementary Materials. Further inquiries can be directed to the corresponding author(s).

## Supplementary Materials

The following supporting information can be downloaded at: https://www.mdpi.com/article/10.3390/antiox14040435/s1, File S1. CONSORT 2010 checklist of information to include when reporting a randomised trial.

## References

[1] Rattan S.I. (2008). Hormesis in aging. Ageing Res. Rev..

[2] Calabrese E.J., Dhawan G., Kapoor R., Iavicoli I., Calabrese V. (2016). HORMESIS: A Fundamental Concept with Widespread Biological and Biomedical Applications. Gerontology.

[3] McCord J.M., Gao B., Hybertson B.M. (2023). The Complex Genetic and Epigenetic Regulation of the Nrf2 Pathways: A Review. Antioxidants.

[4] Hayes J.D., Dinkova-Kostova A.T. (2014). The Nrf2 Regulatory Network Provides an Interface between Redox and Intermediary Metabolism. Trends Biochem. Sci..

[5] He F., Ru X., Wen T. (2020). NRF2, a Transcription Factor for Stress Response and Beyond. Int. J. Mol. Sci..

[6] Onoki T., Izumi Y., Takahashi M., Murakami S., Matsumaru D., Ohta N., Wati S.M., Hatanaka N., Katsuoka F., Okutsu M. (2021). Skeletal muscle-specific Keap1 disruption modulates fatty acid utilization and enhances exercise capacity in female mice. Redox Biol..

[7] Wang L., Yang S., Yan L., Wei H., Wang J., Yu S., Kong A.T., Zhang Y. (2019). Hypoxia preconditioning promotes endurance exercise capacity of mice by activating skeletal muscle Nrf2. J. Appl. Physiol..

[8] Gao L., Kumar V., Vellichirammal N.N., Park S.Y., Rudebush T.L., Yu L., Son W.M., Pekas E.J., Wafi A.M., Hong J. (2020). Functional, proteomic and bioinformatic analyses of Nrf2- and Keap1- null skeletal muscle. J. Physiol..

[9] Martinez-Canton M., Galvan-Alvarez V., Martin-Rincon M., Calbet J.A.L., Gallego-Selles A. (2024). Unlocking peak performance: The role of Nrf2 in enhancing exercise outcomes and training adaptation in humans. Free Radic. Biol. Med..

[10] Cokdinleyen M., Alvarez-Rivera G., Tejera J.L.G., Mendiola J.A., Valdés A., Kara H., Ibáñez E., Cifuentes A. (2024). Tetraselmis chuii Edible Microalga as a New Source of Neuroprotective Compounds Obtained Using Fast Biosolvent Extraction. Int. J. Mol. Sci..

[11] Mantecón L., Moyano R., Cameán A.M., Jos A. (2019). Safety assessment of a lyophilized biomass of *Tetraselmis chuii* (TetraSOD^®^) in a 90 day feeding study. Food Chem. Toxicol..

[12] Ramírez P., Torres S., Lama C., Mantecón L., Unamunzaga C., Infante C. (2020). TetraSOD^®^ activates the antioxidant response pathway in human cells: An in vitro approach. Afr. J. Biotechnol..

[13] Sharp M., Sahin K., Stefan M., Orhan C., Gheith R., Reber D., Sahin N., Tuzcu M., Lowery R., Durkee S. (2020). Phytoplankton Supplementation Lowers Muscle Damage and Sustains Performance across Repeated Exercise Bouts in Humans and Improves Antioxidant Capacity in a Mechanistic Animal. Nutrients.

[14] Sharp M.H., Sahin K., Stefan M.W., Gheith R.H., Reber D.D., Ottinger C.R., Orhan C., Tuzcu M., Sahin N., Lowery R.P. (2021). Marine Phytoplankton Improves Exercise Recovery in Humans and Activates Repair Mechanisms in Rats. Int. J. Sports Med..

[15] Sharp M., Wilson J., Stefan M., Gheith R., Lowery R., Ottinger C., Reber D., Orhan C., Sahin N., Tuzcu M. (2021). Marine phytoplankton improves recovery and sustains immune function in humans and lowers proinflammatory immunoregulatory cytokines in a rat model. Phys. Act. Nutr..

[16] Clifford T., Acton J.P., Cocksedge S.P., Davies K.A.B., Bailey S.J. (2021). The effect of dietary phytochemicals on nuclear factor erythroid 2-related factor 2 (Nrf2) activation: A systematic review of human intervention trials. Mol. Biol. Rep..

[17] Thorley J., Thomas C., Thon N., Nuttall H., Martin N.R.W., Bishop N., Bailey S.J., Clifford T. (2024). Combined effects of green tea supplementation and eccentric exercise on nuclear factor erythroid 2-related factor 2 activity. Eur. J. Appl. Physiol..

[18] Thorley J., Alhebshi A., Rodriguez-Mateos A., Zhang Z., Bailey S.J., Martin N.R.W., Bishop N.C., Clifford T. (2024). Acute supplementation with a curcuminoid-based formulation fails to enhance resting or exercise-induced NRF2 activity in males and females. Food Funct..

[19] Toro V., Siquier-Coll J., Bartolomé I., Robles-Gil M.C., Rodrigo J., Maynar-Mariño M. (2020). Effects of Tetraselmis chuii Microalgae Supplementation on Ergospirometric, Haematological and Biochemical Parameters in Amateur Soccer Players. Int. J. Environ. Res. Public Health.

[20] García Á., Toro-Román V., Siquier-Coll J., Bartolomé I., Muñoz D., Maynar-Mariño M. (2022). Effects of Tetraselmis chuii Microalgae Supplementation on Anthropometric, Hormonal and Hematological Parameters in Healthy Young Men: A Double-Blind Study. Int. J. Environ. Res. Public Health.

[21] Sies H., Jones D.P. (2020). Reactive oxygen species (ROS) as pleiotropic physiological signalling agents. Nat. Rev. Mol. Cell Biol..

[22] Nikolaidis M.G., Margaritelis N.V., Matsakas A. (2020). Quantitative Redox Biology of Exercise. Int. J. Sports Med..

[23] Westerblad H., Allen D.G. (2011). Emerging roles of ROS/RNS in muscle function and fatigue. Antioxid. Redox Signal..

[24] Uhlen M., Oksvold P., Fagerberg L., Lundberg E., Jonasson K., Forsberg M., Zwahlen M., Kampf C., Wester K., Hober S. (2010). Towards a knowledge-based Human Protein Atlas. Nat. Biotechnol..

[25] Livak K.J., Schmittgen T.D. (2001). Analysis of relative gene expression data using real-time quantitative PCR and the 2(−ΔΔC(T)) Method. Methods.

[26] Silver N., Best S., Jiang J., Thein S.L. (2006). Selection of housekeeping genes for gene expression studies in human reticulocytes using real-time PCR. BMC Mol. Biol..

[27] Kuang J., McGinley C., Lee M.J.-C., Saner N.J., Garnham A., Bishop D.J. (2022). Interpretation of exericse-induced changes in human skeletal muscle mRNA expression depends on the timing of the post-exercise biopsies. Peer J..

[28] Bailey S.J., Wilkerson D.P., Dimenna F.J., Jones A.M. (2009). Influence of repeated sprint training on pulmonary O_2_ uptake and muscle deoxygenation kinetics in humans. J. Appl. Physiol..

[29] Whipp B.J., Davis J.A., Torres F., Wasserman K. (1981). A test to determine parameters of aerobic function during exercise. J. Appl. Physiol. Respir. Environ. Exerc. Physiol..

[30] Vomund S., Schäfer A., Parnham M.J., Brüne B., von Knethen A. (2017). Nrf2, the Master Regulator of Anti-Oxidative Responses. Int. J. Mol. Sci..

[31] Pei J., Pan X., Wei G., Hua Y. (2023). Research Progress of Glutathione Peroxidase Family (GPX) in Redoxidation. Front. Pharmacol..

[32] Manevich Y., Fisher A.B. (2005). Peroxiredoxin 6, a 1-Cys peroxiredoxin, functions in antioxidant defense and lung phospholipid metabolism. Free Radic. Biol. Med..

[33] Wadley A.J., Aldred S., Coles S.J. (2016). An unexplored role for Peroxiredoxin in exercise-induced redox signalling?. Redox Biol..

[34] Averill-Bates D.A. (2023). The antioxidant glutathione. Vitam. Horm..

[35] Grillo M.P. (2012). Bioactivation by Phase-II-Enzyme-Catalyzed Conjugation of Xenobiotics. Encyclopedia of Drug Metabolism and Interactions.

[36] Hayes J.D., McLellan L.I. (1999). Glutathione and glutathione-dependent enzymes represent a co-ordinately regulated defence against oxidative stress. Free Radic. Res..

[37] Barbieri E., Sestili P. (2012). Reactive oxygen species in skeletal muscle signaling. J. Signal Transduct..

[38] Murakami A. (2018). Non-specific protein modifications may be novel mechanism underlying bioactive phytochemicals. J. Clin. Biochem. Nutr..

[39] Scuto M., Rampulla F., Reali G.M., Spanò S.M., Trovato Salinaro A., Calabrese V. (2024). Hormetic Nutrition and Redox Regulation in Gut-Brain Axis Disorders. Antioxidants.

[40] L’honoré A., Commère P.H., Negroni E., Pallafacchina G., Friguet B., Drouin J., Buckingham M., Montarras D. (2018). The role of Pitx2 and Pitx3 in muscle stem cells gives new insights into P38α MAP kinase and redox regulation of muscle regeneration. eLife.

[41] Ji L.L., Kang C., Zhang Y. (2016). Exercise-induced hormesis and skeletal muscle health. Free Radic. Biol. Med..

[42] Fittipaldi S., Mercatelli N., Dimauro I., Jackson M.J., Paronetto M.P., Caporossi D. (2015). Alpha B-crystallin induction in skeletal muscle cells under redox imbalance is mediated by a JNK-dependent regulatory mechanism. Free Radic. Biol. Med..

[43] Jeyapaul J., Jaiswal A.K. (2000). Nrf2 and c-Jun regulation of antioxidant response element (ARE)-mediated expression and induction of gamma-glutamylcysteine synthetase heavy subunit gene. Biochem. Pharmacol..

[44] Venugopal R., Jaiswal A.K. (1998). Nrf2 and Nrf1 in association with Jun proteins regulate antioxidant response element-mediated expression and coordinated induction of genes encoding detoxifying enzymes. Oncogene.

[45] Singh V., Ubaid S. (2020). Role of *Silent Information Regulator 1* (*SIRT1*) in Regulating Oxidative Stress and Inflammation. Inflammation.

[46] Baird L., Yamamoto M. (2020). The Molecular Mechanisms Regulating the NRF2 Pathway. Mol. Cell Biol..

[47] Dinkova-Kostova A.T., Holtzclaw W.D., Kensler T.W. (2005). The role of Keap1 in cellular protective responses. Chem. Res. Toxicol..

[48] Reid M.B. (2001). Invited Review: Redox modulation of skeletal muscle contraction: What we know and what we don’t. J. Appl. Physiol..

[49] Zhang D.D., Lo S.C., Sun Z., Habib G.M., Lieberman M.W., Hannink M. (2005). Ubiquitination of Keap1, a BTB-Kelch Substrate Adaptor Protein for Cul3, Targets Keap1 for Degradation by a Proteasome-Independent Pathway. J. Biol. Chem..

[50] Tranah G.J., Barnes H.N., Cawthon P.M., Coen P.M., Esser K.A., Hepple R.T., Huo Z., Kramer P.A., Toledo F.G.S., Zhang X. (2024). Expression of mitochondrial oxidative stress response genes in muscle is associated with mitochondrial respiration, physical performance, and muscle mass in the Study of Muscle, Mobility, and Aging. Aging Cell.

[51] Islam H., Bonafiglia J.T., Turnbull P.C., Simpson C.A., Perry C.G.R., Gurd B.J. (2020). The impact of acute and chronic exercise on Nrf2 expression in relation to markers of mitochondrial biogenesis in human skeletal muscle. Eur. J. Appl. Physiol..

[52] Russell A.P., Foletta V.C., Snow R.J., Wadley G.D. (2014). Skeletal muscle mitochondria: A major player in exercise, health and disease. Biochim. Biophys. Acta.

[53] Islam H., Hood D.A., Gurd B.J. (2020). Looking beyond PGC-1α: Emerging regulators of exercise-induced skeletal muscle mitochondrial biogenesis and their activation by dietary compounds. Appl. Physiol. Nutr. Metab..

[54] Kasiak P., Kowalski T., Rębiś K., Klusiewicz A., Ładyga M., Sadowska D., Wilk A., Wiecha S., Barylski M., Poliwczak A.R. (2024). Is the Ventilatory Efficiency in Endurance Athletes Different?-Findings from the NOODLE Study. J. Clin. Med..

[55] Joyner M.J., Coyle E.F. (2008). Endurance exercise performance: The physiology of champions. J. Physiol..

[56] Kurl S., Hakkarainen P., Voutilainen A., Lönnroos E. (2022). Combined effects of maximal oxygen uptake and glucose status on mortality: The Prospective KIHD cohort study. Scand. J. Med. Sci. Sports.

[57] Ung G.A., Nguyen K.H., Hui A., Wong N.D., Dineen E.H. (2024). Impact of cardiorespiratory fitness and diabetes status on cardiovascular disease and all-cause mortality: An NHANES retrospective cohort study. Am. Heart J. Plus.

[58] Margaritelis N.V., Paschalis V., Theodorou A.A., Kyparos A., Nikolaidis M.G. (2020). Antioxidant supplementation, redox deficiencies and exercise performance: A falsification design. Free Radic. Biol. Med..

